# The effect of germinated black lentils on cookie quality by applying ultraviolet radiation and ultrasound technology

**DOI:** 10.1111/1750-3841.17002

**Published:** 2024-04-05

**Authors:** Hacer Levent, Kübra Aktaş

**Affiliations:** ^1^ Department of Nutrition and Dietetics, Faculty of Health Sciences Karamanoğlu Mehmetbey University Karaman Turkey; ^2^ Department of Gastronomy and Culinary Arts, School of Applied Sciences Karamanoğlu Mehmetbey University Karaman Turkey

**Keywords:** black lentil, cookie, germination, ultrasound, ultraviolet

## Abstract

**Abstract:**

Black lentils contain protein, carbohydrates, dietary fiber, minerals, and vitamins, as well as phytochemicals and various bioactive compounds. Ultraviolet (UV) radiation and ultrasound (US) methods are innovative technologies that can be used to increase the efficiency of the germination process in grains and legumes. To improve the nutritional value and bioactive compounds of the cookies, black lentils germinated by applying UV radiation and US technology were used in the cookie formulation. Before the germination process, UV, US, and their combination (UV+US) were applied, and pretreated and unpretreated germinated black lentil flours were used at a level of 20% in the cookie formulation. The results revealed that pretreatment application increased the total phenolic content and antioxidant activity more than the lentil sample germinated without any treatment. In addition, the pretreatments applied further reduced the amount of phytic acid in black lentils and the lowest phytic acid content was obtained with the UV–US combination. Compared to cookies containing unpretreated germinated black lentil flour, higher L* values and lower a* values were obtained in the cookie samples containing pretreated germinated black lentil flour. Cookies containing all pretreated germinated lentils generally exhibited higher Ca and K content. This study demonstrated that UV radiation and US improved the nutritional value and bioactive components of the germinated black lentil flour and the cookies in which it was used, compared to the black lentils germinated without any treatment.

**Practical Application:**

Pretreatment of black lentils with UV/US application before germination resulted in a greater increase in total phenolic content and antioxidant activity compared to the control sample. The applied pretreatments caused a further decrease in the amount of phytic acid in black lentil samples. Black lentils germinated with the UV+US combination revealed higher Ca, Fe, K, and Mg content compared to the sample germinated without any treatment.

## 1 INTRODUCTION

Food treatments such as soaking, cooking, germination, fermentation, and roasting are used to increase the functionality of legumes. In researches conducted in this field, the advantages of the germination process on the nutritional properties of legumes have been emphasized and the possible use of germinated legumes in different food products such as beverages, sweet products, bakery products, dairy products, pasta, and crackers has been investigated (Atudorei et al., 2021).

Although germination is a traditional process, it has recently begun to attract attention because it causes significant changes in the structure. For example, increasing the digestibility of starch and protein, increasing the amount of water‐soluble vitamins and amino acids, and decreasing the amount of antinutritional factors can be achieved through this process. These various complex physiological and biochemical changes that occur during seed germination and transformation can be affected by internal and external factors. To optimize germination, factors such as temperature, relative humidity, oxygen pressure, and light must be constantly controlled. On the other hand, the applications of new technologies on germination, such as pulsed electric field, high‐pressure processing, ultrasound (US), ozone treatment, ultraviolet (UV), microwave radiation, magnetic field, plasma‐activated water, non‐thermal plasma, and electrolyzed oxidizing water, have been investigated (Avezum et al., 2022; Rifna et al., 2019; Wang et al., 2019).

US treatment is applied with sound waves at frequencies ranging from 20 to 100 kHz and a sound intensity of 10–1000 W/cm^2^ in food applications. The cavitation bubbles that occur act as a series of dilution and compression cycles that disrupt molecules present in the liquid, leading to the rupture of cell walls and progressive mass transfer of cell contents. Studies on this subject elucidated the effects of US on germination by the creation of fissures on the pericarp and seed (Polat et al., 2020; Rifna et al., 2019). US stimulates seed germination, increases the germination percentage, and accelerates the growth of plants (Wang et al., 2012; Yang et al., 2015).

UV is defined as electromagnetic light radiation and can originate from the sun or a light source. UV light is classified according to different wavelengths as UV‐A (315–400 nm), UV‐B (280–315 nm), UV‐C (200–280 nm), and vacuum UV (100–200 nm), and researchers compared the effects of these UV types on selected plant/food characterizations (Rifna et al., 2019). UV light can easily spread into plant tissue due to its high energy, causing morphological, physiological, and biochemical changes in plants. Also, Thomas and Puthur (2017) explained that when different plants such as wheat, rice, corn, cowpeas, and cucumbers are exposed to low levels of UV radiation, seed germination, growth, seed coat thickness, and biomass are stimulated, and photosynthesis and pigment content are increased.

Cookies are among the most preferred baked goods due to their easy accessibility, long shelf life, and low cost, and the market size is increasing day by day. The world production of biscuits is 18.95 million tons (Leonel et al., 2021). The biscuit market reached $76.385 billion at the end of 2017 and is expected to reach $164 billion by 2024, at a compound annual growth rate of 5.08% (Goubgou et al., 2021; Kaur et al., 2023). However, it is commonly produced from refined wheat flour and has high fat and sugar and low protein content (Yang et al., 2022).

To the best of our knowledge, there is no research in the literature on the application of UV radiation and US technology in black lentil germination. Therefore, the objectives of this study were to (1) determine the effects of UV radiation and US technology on the nutritional composition of germinated lentils, separately and in combination; (2) improve the nutritional quality of cookies by using germinated black lentil flours; (3) evaluate the physical, chemical, and technological quality of cookies prepared with partial substitution of pretreated and unpretreated germinated black lentil flours.

## 2 MATERIALS AND METHODS

### 2.1 Materials

Ingredients in cookie formulation; commercial refined wheat flour (Hekimoğlu Milling Industry and Trade Inc.), shortening (Besler Food and Chemical Industry and Trade Inc.), sodium bicarbonate, powdered sugar and ethyl vanillin (Dr. Oetker Food Industry and Trade Inc.), salt (Billur Salt Trade Inc.), non‐fat dry milk (Enka Dairy Inc.), and black lentils (Yayla Pulses Inc.) were purchased from local markets in Karaman (Turkey).

### 2.2 Germination of black lentils

The germination process was applied by traditional and non‐traditional methods. To obtain traditionally germinated black lentils, sodium hypochlorite (NaClO) solution was used for disinfection, and then the lentils, which were soaked in water for 3 h, were spread on cotton sheets. Black lentils were moistened every 12 h and allowed to germinate in a dark place at 25 ± 2°C for 96 h. The cabinet conditions were 20  ±  2°C and 80%−90% relative humidity. This black lentil sample was called “germinated black lentils (GBL) (without pretreatments).” UV, US, and the combination of these processes (UV+US) were applied as pretreatments to obtain non‐traditional germinated black lentils. Black lentils were exposed to UV light (365 nm, approximately 372 W/m^2^) for 30 min in a lab‐made photoreactor (Kerman UV/18) containing 18 Philips TL 8 W BLB lamps (Guven et al., 2020). On the other hand, an ultrasonic bath (Bandelin Electronic GmbH&Co., Berlin, Germany; 35 kHz, 160–640 W) was used for US application, and black lentils were exposed to US at 35 kHz frequency in pure water at 20°C for 10 min. For the combined application, US was first applied to the black lentils for 10 min, and then UV was applied for 30 min (Kibar, 2020). These lentils were entitled “UV‐assisted germinated black lentils (GBL),” “US‐assisted germinated black lentils (GBL),” and “UV+US‐assisted germinated black lentils (GBL),” respectively. All germinated and raw black lentils were dried in an oven (Nüve FN‐500, Ankara, Turkey) at 50°C and ground into whole grain flour (<  500 µm) using a laboratory mill (Bosch MKM600, İstanbul, Turkey). The analyses were conducted with lentil powders.

### 2.3 Preparation of cookies

The modified method of the American Association of Cereal Chemists (AACC) with the number 10–54.01 was used for cookie production (AACC, 1990). The formulation used consists of wheat flour (100 g), shortening (40 g), powdered sugar (40 g), salt (1 g), ethyl vanillin (0.5 g), sodium bicarbonate (2 g), non‐fat dry milk (1 g), and water (15–25 mL). The amount of water was determined by preliminary trials according to the optimum dough consistency. For the production of enriched cookies, wheat flour in the formulation was replaced with 20% black lentil flour. All ingredients were mixed with a laboratory mixer for 10 min. The obtained dough was laminated to a thickness of 5 mm and shaped using a circular dough cutter (inner diameter: 50 mm). The dough pieces were baked in an electric oven for 18 min at 160°C. Cookie samples were cooled and stored in glass airtight containers at room temperature until used for analysis.

### 2.4 Physical and mechanical properties

The Hunter color values (L* [lightness], a* [greenness/redness], and b* [blueness/yellowness]) were measured with a Minolta colorimeter (Konica CR 400, Japan). Saturation index (SI) and hue values were calculated using a* and b* parameters according to the equations [(a*2+b*2)^1/2^] and [arctan (b*/a*)], respectively. All measurements were made at three different points. Diameter and thickness measurements of the cookies were made using a digital micrometer (0.001 mm precision degree). Then, spread ratio values were found by dividing the diameter by the thickness. The hardness and fracturability of the cookie samples were determined with a texture analyzer (TA‐XT2, Stable Microsystems, Surrey, UK). Three‐point bend test was performed and test conditions were: test mode: compression; trigger force: 50 g; load cell: 5 kg; pre‐test, test, and post‐test speed: 1.00, 3.00, and 10.0 mm/s; distance: 5 mm.

### 2.5 Chemical composition

AACC methods numbered 44‐19, 08‐01, 46‐12, and 30‐25 were applied to determine moisture, ash, crude protein, and crude fat contents, respectively (AACC, 1990). The phytic acid content of the samples was determined using the colorimetric method proposed by Haugh and Lantzsch (1983). For this purpose, HCl was used for phytic acid extraction, and then Fe(III) solution was used for phytic acid precipitation. Iron concentrations in the extracts were determined by spectrophotometry. The amount of phytic acid was expressed as mg/100 g. The same extraction method was used to determine both the total phenolic content (TPC) and antioxidant activities (AA) of the samples. The samples were extracted with 10 mL of solution consisting of a mixture of hydrochloric acid, methanol, and distilled water at a ratio of 1/8/1 v/v, respectively, for 2 h at room temperature (Beta et al., 2005; Gao et al., 2002). Folin–Ciocalteu reagent and colorimetric method were used for TPC estimation. After centrifugation at 1600 g for 10 min, 0.1 mL of extract, 0.5 mL of Folin–Ciocalteu reagent (10% diluted, v/v, water), and 1.5 mL of sodium carbonate solution (20%, w/v) were mixed in the test tube and filled to 10 mL with distilled water. The mixture was kept in the dark and at ambient temperature for 120 min. Absorbance measurements were performed with a spectrophotometer (Shimadzu UV1800, Japan) at 760 nm, and the results are expressed as gallic acid equivalents (GAE) per kg of sample on a dry matter basis. Also, DPPH (2‐2‐diphenyl‐2‐picrylhydrazyl) and colorimetric methods were used for AA estimation (Beta et al., 2005; Gyamfi et al., 1999). Absorbance measurements were performed at 517 nm at 0 and 30 min. The results were calculated according to the formula:

$$\begin{array}{ccc} & & \mathrm{Inhibition}\;\%=\\ & & \left[\left(A\;\mathrm{of}\;\mathrm{Contro}{l_{t}}_{=0}-A\;\mathrm{of}\;\mathrm{Sampl}{e_{t}}_{=30}\right)/\left.A\;\mathrm{of}\;\mathrm{Contro}{l_{t}}_{=0}\right)\right]\\ & & \times \;100. \end{array}$$

Ca, Fe, K, Mg, and Zn contents of the samples were determined. The powdered samples were burned with nitric acid. The samples were then treated with perchloric acid and heated for 5–6 h. The samples were cooled to room temperature and hydrogen peroxide was added and reheated until discoloration was observed. ICP‐OES (inductively coupled plasma optical emission spectrometry, Agilent 720, USA) was used for the determination of the mineral contents of filtrates (Levent et al., 2020).

### 2.6 Statistical analysis

Results are shown as mean with standard deviation and compared using analysis of variance. SPSS statistical software (IBM SPSS Statistics version 19, 2010) was used to analyze the data. Multiple comparisons of means were made by Duncan's procedure, and statistical significance was considered at *p* < 0.05 level.

## 3 RESULTS AND DISCUSSION

### 3.1 Physical and chemical properties of raw materials

The color values and chemical composition of five different black lentils as well as wheat flour were presented in Table 1. Black lentil samples had lower L^*^ values (57.46–67.91) compared with wheat flour (93.23), as expected, and the lowest L^*^ value was obtained with UV‐assisted GBL. There was no significant difference between the a* values of different GBL samples (1.48–1.74), and these values were higher than those of raw lentil samples (0.34). In general, b* and SI values of raw lentil and US‐assisted GBL samples were found to be higher than the others. When the raw materials were evaluated in terms of *hue* value, the highest value was determined in wheat flour (92.86), and the lowest values were determined in the GBL without any treatment (82.96) and UV‐assisted GBL (82.80) samples. According to chemical analysis, no significant difference was determined between the crude protein, ash, and crude fat contents of the GBL samples. While the ash, crude protein, and crude fat contents in the raw lentil sample were determined as 3.89%, 26.20%, and 1.06%, respectively, these values were found in the ranges of 4.26%–4.41%, 27.36%–28.10%, and 3.18%–3.40% in the GBL samples, respectively. The ash and protein contents of raw lentils increased slightly with all applications carried out together with the germination process. Dry matter loss from the breakdown of carbohydrates in germination causes an increase in the amount of protein and dietary fiber contents (Malleshi & Klopfenstein, 1998). The germination process significantly affected the TPC results (*p* < 0.05), and a similar increase was observed in the AA results. While the TPC value was 2574.62 mg GAE/kg in the raw lentil sample, this value increased to 3352.40, 3436.15, 3497.69, and 3517.50 mg GAE/kg in the GBL (without pretreatments), UV‐assisted, US‐assisted, and UV+US‐assisted GBL samples, respectively. Black lentil samples that were germinated with pretreatment applications had higher AA values compared to the samples germinated without any treatment. Yaver (2022) used immature, mature, fermented, and germinated black chickpea flours at a 20% ratio in bread formulation. It was reported that bread containing germinated and immature black chickpea flour presented the highest TPC among the bread samples. Similarly, Guo et al. (2012) reported that germination dramatically increased the total flavonoids, total phenolic compounds, vitamin C content, and antioxidant activity of mung beans. Yang et al. (2015) reported that US treatment increased the germination rate and length of soybean sprouts and that US‐treated soybeans were a potent source of bioactive compounds such as isoflavones and amino acids. Germination application reduced the phytic acid content of black lentil samples as expected. The lowest and highest phytic acid contents were determined in wheat flour (185.20 mg/100 g) and raw lentil samples (1367.10 mg/100 g). The phytic acid contents of GBL (without pretreatments), UV‐assisted, US‐assisted, and UV+US‐assisted GBL samples were 1077.99, 1031.79, 733.60, and 695.41 mg/100 g, respectively. A lower amount of phytic acid was determined in GBL samples using UV, US, and UV+US combination compared to samples germinated without any treatment. Earlier studies have reported that the phytic acid content of legumes decreased with germination (Mubarak, 2005). Such decreases are due to increased phytase activity during germination. With increased phytase activity, phytates are hydrolyzed and soluble minerals and proteins are released (Demir & Bilgiçli, 2020). Since the application of US and UV or UV–US increased the germination efficiency, it may have reduced the amount of phytic acid more than the black lentils germinated without any treatment.

**TABLE 1:** Physical and chemical properties of raw materials.

	WF	BL (Raw)	GBL (without pretreatments)	UV‐assisted GBL	US‐assisted GBL	UV+US‐assisted GBL
L*	93.23 ± 0.24a	62.56 ± 0.31c	67.91 ± 0.28b	57.46 ± 0.40e	59.95 ± 0.21d	60.54 ± 0.18d
a*	−0.50 ± 0.07c	0.34 ± 0.10b	1.71 ± 0.06a	1.74 ± 0.04a	1.48 ± 0.11a	1.49 ± 0.07a
b*	10.02 ± 0.31d	16.29 ± 0.25a	13.84 ± 0.41c	13.77 ± 0.16c	15.89 ± 0.27ab	14.85 ± 0.18bc
SI	10.03 ± 0.36d	16.29 ± 0.27a	13.95 ± 0.18c	13.88 ± 0.25c	15.96 ± 0.13a	14.92 ± 0.20b
Hue	92.86 ± 0.36a	88.80 ± 0.20b	82.96 ± 0.15d	82.80 ± 0.42d	84.68 ± 0.28c	84.27 ± 0.35c
Moisture (g/100 g)	9.89 ± 0.24a	4.96 ± 0.16c	5.87 ± 0.27b	5.84 ± 0.20b	5.63 ± 0.11bc	5.27 ± 0.21bc
Ash (g/100 g)	0.6 ± 0.07c	3.89 ± 0.13b	4.26 ± 0.16ab	4.41 ± 0.18a	4.35 ± 0.06ab	4.37 ± 0.11ab
Crude protein(g/100 g)	9.85 ± 0.20c	26.20 ± 0.57b	28.10 ± 0.42a	27.48 ± 0.33ab	27.36 ± 0.20ab	27.64 ± 0.23a
Crude fat (g/100 g)	1.07 ± 0.17b	1.06 ± 0.21b	3.40 ± 0.28a	3.26 ± 0.35a	3.18 ± 0.18a	3.31 ± 0.16a
TPC (mg GAE/kg)	859.00 ± 14.85e	2574.62 ± 10.58d	3352.40 ± 8.10c	3436.15 ± 9.33b	3497.69 ± 6.92a	3517.50 ± 5.11a
AA (Inhibition %)	10.66 ± 0.23d	31.44 ± 0.38c	40.87 ± 0.27b	42.80 ± 0.42a	43.61 ± 0.30a	43.88 ± 0.47a
Phytic acid (mg/100 g)	185.20 ± 6.79f	1367.10 ± 9.05a	1077.99 ± 6.35b	1031.79 ± 4.79c	733.60 ± 5.94d	695.41 ± 7.51e
Ca(mg/100 g)	28.74 ± 0.25f	75.82 ± 0.45e	158.90 ± 0.33c	162.10 ± 0.57b	152.75 ± 0.40d	166.53 ± 0.35a
Fe (mg/100 g)	1.24 ± 0.13d	7.52 ± 0.17c	8.35 ± 0.21b	8.86 ± 0.16ab	8.60 ± 0.28ab	9.13 ± 0.18a
K (mg/100 g)	61.95 ± 0.41e	79.04 ± 0.27d	122.94 ± 0.33c	139.52 ± 0.54ab	138.70 ± 0.41b	140.36 ± 0.37a
Mg (mg/100 g)	27.70 ± 0.28e	68.64 ± 0.34d	75.48 ± 0.38c	75.58 ± 0.25b	77.50 ± 0.57ab	79.20 ± 0.31a
Zn (mg/100 g)	0.90 ± 0.08c	2.04 ± 0.17b	2.76 ± 0.07a	2.88 ± 0.04a	2.85 ± 0.13a	2.91 ± 0.08a

Abbreviations: WF, wheat flour; BL, black lentil; GBL, germinated black lentil; UV, ultraviolet; US, ultrasound; TPC, total phenolic content; AA, antioxidant activity.

Means followed by different letters within a row are significantly different (*p* < 0.05). Values are the average of triplicate measurements on the duplicate samples. Chemical properties except moisture are based on dry matter.

While the mineral content of all lentil samples was found to be higher than wheat flour, the germination process also increased the mineral content of raw lentil samples. High mineral contents were obtained especially by applying the US‐UV combination as a pretreatment. Ca, Fe, K, Mg, and Zn contents of black lentils increased by 109.6%, 11.0%, 55.5%, 9.96%, and 35.3%, respectively, with germination without pre‐treatments. However, the Ca, Fe, K, Mg, and Zn contents of black lentils germinated by applying the UV+US combination increased by 119.6%, 21.4%, 77.6%, 15.4%, and 42.6%, respectively. In other words, germination with the application of UV–US caused an extra increase in the Ca, Fe, K, and Mg of black lentils compared to the lentil sample germinated without pre‐treatment. The decrease in the amount of phytic acid with germination was mostly obtained with UV–US application, followed by US and UV, respectively (Table 1). The decrease in the amount of phytic acid may have caused less complex formation in minerals. Wang et al. (2015) reported that phosphatase and phytase activities of germinated mung beans and soybeans were increased compared to raw beans. This may cause the complex structures formed by phytic acid to be hydrolyzed and minerals to be released. In addition, as mentioned before, the use of carbohydrates in the germination process causes a proportional change in the chemical composition, which is another factor affecting the change in mineral amounts (Kömürcü & Bilgiçli, 2023).

### 3.2 Physical properties of cookie samples

The color characteristics of the cookies are shown in Table 2. The substitution of wheat flour with black lentils in the formulation decreased L^*^ values and increased a^*^ values. This may be because the L* value of wheat flour, the main raw material, is higher than that of black lentil flour (Table 1). L* and a* values of cookies with raw black lentils (CRBL) and cookies with germinated black lentils (without pretreatments, CGBL) were found to be statistically similar. Generally, higher L* and lower a* values were obtained in cookies with UV and US applications compared to CRBL and CGBL. On the other hand, cookies with US‐assisted GBL (CUS) and cookies with UV+US‐assisted GBL (CUVS) gave lower b* and SI values compared to other samples. In cookie samples, SI and Hue values changed between 24.24 and 28.30 and 80.80 and 88.12, respectively. CGBL had the lowest *hue* values among cookie samples. In addition to the color values of the raw materials used in cookies, color changes may occur due to the Maillard reaction occurring more in cookies containing black lentil flour due to their high protein content.

**TABLE 2:** Color values of cookie samples.

	L*	a*	b*	SI	Hue
CC	76.93 ± 0.49a	0.89 ± 0.17d	27.17 ± 0.21a	27.18 ± 0.19b	88.12 ± 0.18a
CRBL	64.64 ± 0.34d	4.06 ± 0.11a	28.01 ± 0.16a	28.30 ± 0.37a	81.75 ± 0.22c
CGBL	64.93 ± 0.18d	4.47 ± 0.08a	27.60 ± 0.35a	27.96 ± 0.16ab	80.80 ± 0.14d
CUV	67.30 ± 0.35c	3.57 ± 0.13b	27.49 ± 0.11a	27.72 ± 0.23ab	82.60 ± 0.17c
CUS	69.44 ± 0.41b	1.91 ± 0.06c	24.16 ± 0.28b	24.24 ± 0.11c	85.48 ± 0.24b
CUVS	67.13 ± 0.37c	2.17 ± 0.10c	24.17 ± 0.23b	24.27 ± 0.17c	84.87 ± 0.31b

Abbreviations: SI, saturation index; CC, control cookie; CRBL, cookies with raw black lentil; CGBL, cookies with germinated black lentil; CUV, cookies with UV‐assisted germinated black lentil; CUS, cookies with US‐assisted germinated black lentil; CUVS, cookies with UV+US‐assisted germinated black lentil.

Means followed by different letters within a column are significantly different (*p* < 0.05). Values are the average of triplicate measurements on the duplicate samples.

**FIGURE 1:**
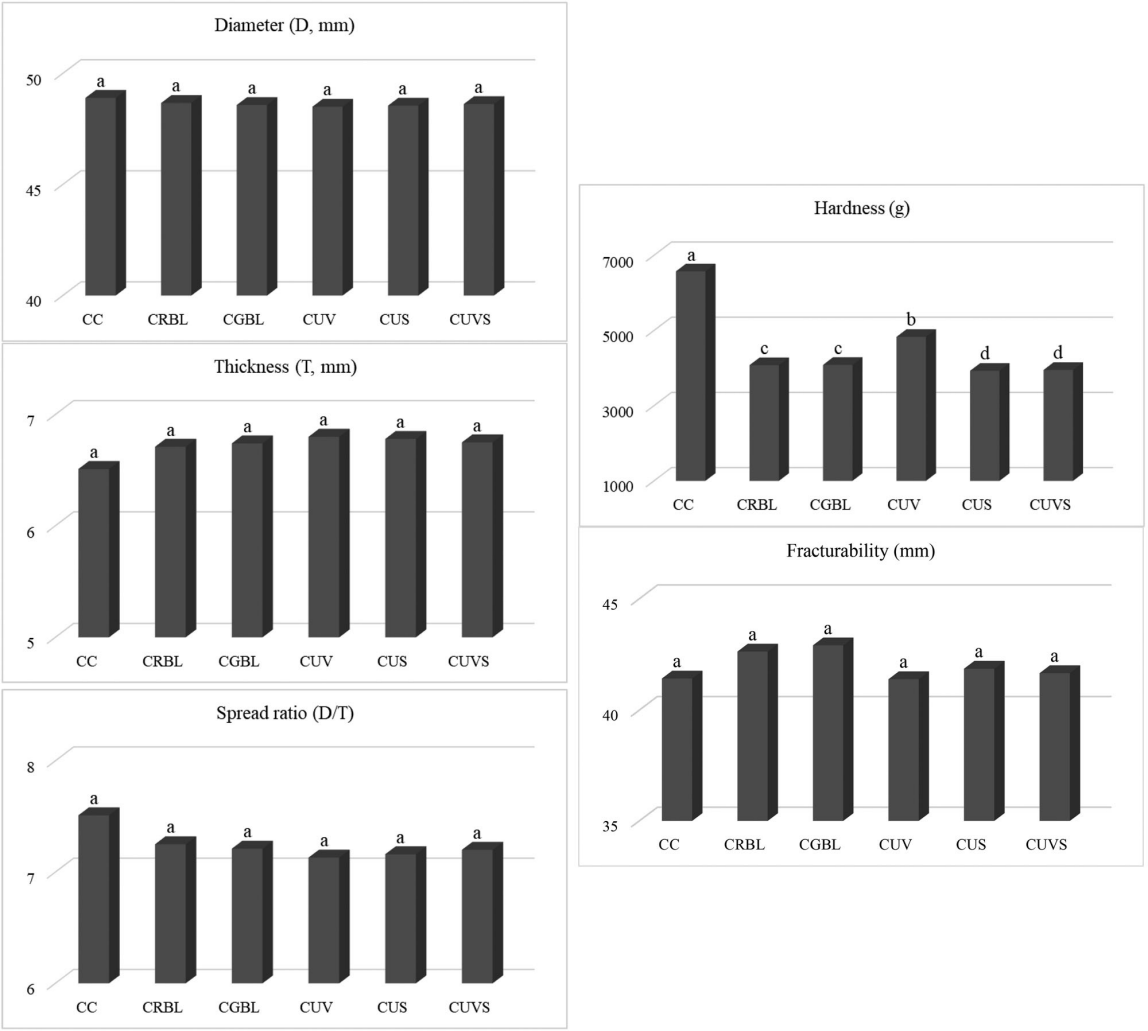
Diameter, thickness, spread ratio, hardness, and fracturability values of cookie samples. CC, control cookie; CRBL, cookies with raw black lentil; CGBL, cookies with germinated black lentil; CUV, cookies with UV‐assisted germinated black lentil; CUS, cookies with US‐assisted germinated black lentil; CUVS, cookies with UV+US‐assisted germinated black lentil.

The diameter, thickness, spread ratio, hardness, and fracturability values of the cookie samples are given in Figure 1. The diameter and thickness values of cookies were close to each other, and there were no significant differences in results. The diameter and thickness values ranged between 48.50 and 48.90 mm and 6.51 and 6.80 mm, respectively. As a result of this situation, the changes in the spread ratio results were not found to be significant (*p* < 0.05). Additionally, hardness values exhibited significant differences and the use of black lentils led to decreases. After the control cookie (CC), the highest hardness value was obtained in cookies with UV‐assisted GBL (CUV), while the lowest values were found in CUS and CUVS. Hardness was determined as 6575.33, 3927.98, and 3950.86 g in control, CUS, and CUVS, respectively. Fracturability values of cookie samples ranged between 41.39 and 42.91 mm, and no significant difference was found between them. Chung et al. (2014) reported the reduction in gluten content by using composite flour in cookie formulation resulted in retarding the formation of gluten matrices, which contributed to the substantial decrease in hardness. Oskaybaş‐Emlek et al. (2021) used raw and germinated lentil flour in cookie production and reported that the germinated lentil flour cookie exhibited the lowest hardness values among cookie samples.

### 3.3 Chemical properties of cookie samples

According to the statistical test results shown in Table 3, the use of black lentils with or without different processes had no significant effect on the moisture, ash, and crude fat contents of cookies. Although the use of black lentils in the formulation increased the protein content compared to the control, no significant difference was observed between the protein contents of the samples containing black lentils. The lowest protein value was determined in the CC (7.61%), while protein values in cookies containing black lentils varied between 9.98% and 10.80%. Similarly, Singh Sibian and Singh Riar (2021) reported that the use of high‐protein legume flour and germination of grains increased the total protein content of composite flour cookies by approximately 50% compared to the control (wheat flour cookies). Lentil seeds have a rich nutritional content, especially protein, and provide sufficient amounts of essential amino acids when consumed with cereals (Kamboj & Nanda, 2018). Even though phytic acid has shown positive health and physiological effects because of its antioxidant activity, it is commonly known as an anti‐nutrient due to the food micronutrient chelating ability, making them unabsorbable and thus of low bioavailability (Bloot et al., 2023). The phytic acid contents of the cookie samples varied between 218.40 and 458.20 mg/100 g, and the lowest and highest values belonged to the CC and CRBL samples, respectively. On the other hand, the germination process reduced the phytic acid content, and lower values (312.27 and 305.40 mg/100 g) were obtained, especially in CUS and CUVS. Wang et al. (2015) reported that phytic acid contents decreased by 57.5% in germinated soybeans and by 76.0% in mung beans, and the bioavailability of Fe and Zn increased. Rasha Mohamed et al. (2011) investigated the effect of processing treatments including dehulling, soaking, autoclaving, boiling, microwave cooking, fermentation, and germination on the phytic acid content of mung bean, soybean, and kidney bean. It was reported that germination and fermentation individually or in combination with cooking and dehulling processes caused significant (*p* < 0.05) decreases in phytic acid content, more than the other processing treatments. TPC was found to be higher in samples containing germinated black lentils compared to CC (859.29 mg GAE/kg) and CRBL (1083.08 mg GAE/kg). CUS and CUVS samples showed higher TPC values compared to CGBL (1330.27 mg GAE/kg). A similar trend was observed in AA values. Higher AA results were obtained in samples containing germinated black lentils, and the results ranged between 10.76% and 19.34%. These results agree well with those reported by Oskaybaş‐Emlek et al. (2021). In the study conducted by Khang et al. (2016), individual phenolic and antioxidant capacities of six legumes during germination were examined, and it was determined that the phenolic content of all legumes increased significantly during germination.

**TABLE 3:** Chemical properties of cookie samples.

	Moisture (g/100 g)	Ash (g/100 g)	Crude protein (g/100 g)	Crude fat (g/100 g)	Phytic acid (mg/100 g)	TPC (mg GAE/kg)	AA (inhibition %)
CC	3.52 ± 0.14a	1.38 ± 0.18a	7.61 ± 0.16b	16.25 ± 0.21a	218.40 ± 8.49d	859.29 ± 5.64d	10.76 ± 0.57d
CRBL	3.20 ± 0.25a	1.46 ± 0.21a	9.98 ± 0.24a	16.37 ± 0.35a	458.20 ± 9.19a	1083.08 ± 7.33c	14.20 ± 0.23c
CGBL	3.42 ± 0.17a	1.50 ± 0.30a	10.71 ± 0.06a	16.48 ± 0.23a	416.55 ± 5.98b	1330.27 ± 10.47b	17.55 ± 0.34b
CUV	3.35 ± 0.20a	1.57 ± 0.23a	10.78 ± 0.17a	16.90 ± 0.41a	408.80 ± 8.41b	1358.92 ± 9.26ab	17.80 ± 0.24b
CUS	3.27 ± 0.18a	1.53 ± 0.21a	10.73 ± 0.25a	16.60 ± 0.27a	312.27 ± 9.35c	1366.50 ± 10.63a	18.12 ± 0.31ab
CUVS	3.30 ± 0.13a	1.48 ± 0.25a	10.80 ± 0.28a	16.95 ± 0.34a	305.40 ± 7.44c	1380.69 ± 6.92a	19.34 ± 0.40a

Abbreviations: TPC, total phenolic content; AA, antioxidant activity; CC, control cookie; CRBL, cookies with raw black lentil; CGBL, cookies with germinated black lentil; CUV, cookies with UV‐assisted germinated black lentil; CUS, cookies with US‐assisted germinated black lentil; CUVS, cookies with UV+US‐assisted germinated black lentil.

Means followed by different letters within a column are significantly different (*p* < 0.05). Values are the average of triplicate measurements on the duplicate samples. Chemical properties except moisture are based on dry matter.

**TABLE 4:** Mineral contents of cookie samples (mg/100 g).

	Ca	Fe	K	Mg	Zn
CC	30.05 ± 0.28d	1.10 ± 0.18b	103.37 ± 0.38d	11.30 ± 0.28c	0.80 ± 0.07b
CRBL	35.90 ± 0.40c	1.73 ± 0.16ab	112.40 ± 0.42c	14.07 ± 0.33b	0.96 ± 0.06ab
CGBL	38.31 ± 0.23b	2.17 ± 0.23a	130.52 ± 0.23b	15.12 ± 0.16ab	1.01 ± 0.10ab
CUV	40.02 ± 0.49a	2.31 ± 0.10a	135.60 ± 0.28a	15.30 ± 0.25a	1.12 ± 0.08ab
CUS	39.62 ± 0.30ab	2.28 ± 0.24a	135.75 ± 0.40a	15.22 ± 0.21a	1.08 ± 0.07ab
CUVS	39.96 ± 0.35a	2.36 ± 0.21a	136.24 ± 0.31a	15.37 ± 0.38a	1.16 ± 0.11a

Abbreviations: CC, control cookie; CRBL, cookies with raw black lentil; CGBL, cookies with germinated black lentil; CUV, cookies with UV‐assisted germinated black lentil; CUS, cookies with US‐assisted germinated black lentil; CUVS, cookies with UV+US‐assisted germinated black lentil.

Means followed by different letters within a column are significantly different (*p* < 0.05). Values are the average of triplicate measurements on the duplicate samples.

When the mineral contents of cookies were examined (Table 4), CC had the lowest values. Ca, Fe, K, Mg, and Zn contents (mg/100 g) of the cookies were found in the range of 30.05–40.02, 1.10–2.36, 103.37–136.24, 11.30–15.37, and 0.80–1.16, respectively. Significant increases were determined in the Ca and K amounts of cookies with the use of germinated black lentil flour compared to raw black lentil flour. On the other hand, although the mineral contents of cookie samples containing pretreated germinated black lentils were found to be statistically the same, the highest mineral values were generally obtained in CUVS.

The Ca, Fe, K, Mg, and Mn contents of CUVS were 4.3%, 8.8%, 4.4%, 1.7%, and 14.9% higher than those of CGBL. The combination of UV radiation and US technology may have affected the germination efficiency of the seed more positively, and this may also be reflected in the mineral content of the cookie. Atudorei et al. (2020) investigated mineral changes in different legume types during the germination process, and it was reported that especially calcium and iron increased for lentil seeds with advanced germination. They pointed out that the amount of phytic acid decreases during germination, and thus, the minerals in bound form are released during germination. They also stated that calcium is sometimes found bound to α‐amylases and that amylases are activated during germination.

## 4 CONCLUSION

The results obtained from this study showed that UV and US methods and their combination can be applied as a pretreatment in black lentil germination. Thanks to these methods, the nutritional content of black lentils increased more than lentils germinated without any treatment, and no negative effects of the combination of these methods were observed.

Especially, the TPC and AA values of the cookies were further increased and the phytic acid content, which is an anti‐nutritional factor, was further reduced. Future studies may investigate the effects of US, UV, and their combinations on the germination of different legumes under different conditions to further increase the nutritional value.

## AUTHOR CONTRIBUTIONS

**Hacer Levent**: Investigation; resources; methodology; formal analysis; writing—review and editing. **Kübra Aktaş**: Investigation; formal analysis; resources; writing—original draft; writing—review and editing.

## CONFLICT OF INTEREST STATEMENT

The authors have no conflict of interest to declare that are relevant to the content of this article.

## Data Availability

The data that support the findings of this study are available from the corresponding author upon reasonable request.
